# Does It Matter Who Exhibits More Green Purchase Behavior of Cosmetic Products in Asian Culture? A Multi-Group Analysis Approach

**DOI:** 10.3390/ijerph17145258

**Published:** 2020-07-21

**Authors:** Farzana Quoquab, Azila Jaini, Jihad Mohammad

**Affiliations:** 1 Azman Hashim Internal Business School (AHIBS), Universiti Teknologi Malaysia, Kuala Lumpur 54100, Malaysia; fqbhabib@ibs.utm.my; 2 Faculty of Entrepreneurship and Business, University Malaysia Kelantan, Pengkalan Chepa 16100, Malaysia; azila.j@umk.edu.my; 3 Department of Management and Marketing, College of Business and Economics, Qatar University, Doha 2713, Qatar

**Keywords:** green purchase behavior, value orientation, gender, pro-environmental belief, personal norm, cosmetics industry

## Abstract

This study attempts to investigate the moderating effect of gender on value-belief-norm relationships. In addition, this study aims to investigate the factors that affect green purchase behavior of cosmetics products. Particularly, this study investigates the causal relationships between values and pro-environmental beliefs, pro-environmental beliefs and personal norms and personal norms and green purchase behavior. An online survey was carried out which yielded 240 usable responses among which 79 responses were obtained from males and 161 from females. Data were analyzed using structural equation modeling, partial least square (PLS-SEM) approach and multi-group analysis (MGA) technique. Results revealed that all direct relationships were supported by the data. It was also found that gender moderates the relationships between altruistic values and pro-environmental beliefs, pro-environmental beliefs and personal norms and personal norms and green purchase behavior. Nevertheless, gender did not moderate the link between hedonic value and pro-environmental beliefs. This study contributes to the existing literature by considering gender as a moderator, which is comparatively new in the green purchase behavior literature. In addition, this study examines few new linkages: more specifically, incorporating hedonic value in value-belief link and adapting value-belief-norm (VBN) theory in measuring consumers’ green purchase behavior.

## 1. Introduction

Green purchase behavior (GPB) can be considered as one of the major contributors towards environmental sustainability [1]. In recent years, consumers became more aware about the green purchase behavior by considering the environmental welfare and quality of life [2]. Certainly, it mobilized the production and sales of green products worldwide. Globally, the sale of green products has increased from $209 billion in 2011 to $845 billion in 2015 [3]. This growth can also be seen in the cosmetics industry [4]. 

It is reported that the Asian market of cosmetics has become one of the fastest growing markets [5]. The market value of the Asia Pacific region has increased to more than US$70 billion. The Malaysian local cosmetics and toiletries market is valued at about MYR 3 billion, with a growth rate of 13% annually [6]. Cosmetic products are made up of a mixture of chemical compounds (e.g., synthetic ingredients). The continuous usage of such kind of cosmetics is likely to harm consumers’ health in the long run and may create long term side effects, such as headaches, acne, hair problems, cancer, skin allergies and other serious illnesses that may affect the life of the consumers [7]. Therefore, there is a need to have a paradigm shift from conventional cosmetics purchase to green cosmetics purchase to minimize the risk on consumers’ health, as well as to reduce its impact on the environment [8].

Based on the report by the Ministry of Health Malaysia, a total of 142,777 illegal cosmetics worth RM 3.4 million were confiscated in 2018 [5]. This phenomenon called for the research attention. Prior studies found that having awareness on environmental issues does not lead to green purchase behavior all the time [9,10]. Some consumers still refuse to purchase green products even though they understand the benefits of adopting green purchase behavior [11]. It is also evident that, though consumers hold positive attitude towards environment, it does not lead to environmentally significant behavior [12,13], which is also true for the cosmetics industry. From the perspective of cosmetic purchase, most consumers randomly choose their preferred cosmetics without realizing or understanding the negative side of the usage [14]. Thus, it is certainly important to investigate factors that may drive consumers’ green purchase behavior [2,5]. Considering the importance of adopting green purchase behavior in the cosmetics industry, the present study aims to examine the factors that can affect consumers’ green purchase behavior in the context of Malaysian cosmetics industry.

This study considered the value-belief-norm (VBN) theory as the theoretical basis. Stern et al. [15] developed the VBN theory to explain the link between human values and their behavior in the environmental context. This theory suggests causal relationships between values, beliefs, norms and behaviors [15]. The relationship between value orientation (biospheric, altruistic and egoistic), belief and personal norm have been investigated in the literature [16]. Nevertheless, less attention was given to the role of hedonic values. Besides, the studies on consumers’ green purchase behavior in different research contexts have been examined [12,17], but limited studies focused on green cosmetic purchase. Hedonic value reflects the value received from the emotive aspects of the buying experience of product and/or services [18]. If consumers perceive purchasing cosmetic products as fun, pleasurable, relaxing, enjoyable, etc., they are more likely to develop positive attitude and ultimately purchase the green product. Therefore, considering the hedonic value is crucial factor that may reflect a clear picture of consumers’ experience of shopping [19]. Accordingly, the first objective of this study is developed: to examine the causal links between values (altruistic and hedonic), perceived environmental belief, personal norm and green purchase behavior in the cosmetic industry in Malaysian context. 

Although consumers’ values, beliefs and norm are likely to exert positive and significant effect on their purchasing behavior of green cosmetic products, these effects may vary for males and females in regards to emotional and psychological attachment and behavioral characteristics of each gender [20]. Nevertheless, few studies have investigated the causal relationships between consumers’ values and their purchase behavior through their beliefs and norm across gender. Perryman et al. [21] argued that gender is an important personal characteristic that influences individuals’ decisions and behaviors. In support of this view, Baker and Ozaki [22] found that gender is an important demographic predictor of consumers’ green purchase behaviors. Similarly, Roberts [23] reported that the majority of the green consumers are females. Likewise, Oztek and Cengel [24] revealed that female consumers are more inclined to behave pro-environmentally compared to male consumers. Another stream of studies found that males have more tendency to participate in pro-environmental behavior compared to females [25,26], while others found no gender differences in performing environmentally significant behavior [27,28]. One possible remedy to solve these inconsistent findings pertaining to consumers’ demographic profile is to consider such variables as moderators [3,29]. Hence, this study considers gender as the major differentiating factor to understand consumers’ green purchase behaviors. Thus, the second objective of this study is formulated: to examine the moderating role of gender among the relationships outlined in the VBN theory.

The rest of the paper is organized as follows: Firstly, relevant literature is reviewed and the conceptual framework is developed. Next, the adapted methodology is discussed followed by the results, findings and discussion. Lastly, a conclusion is made and implications, limitations and future research directions are highlighted. 

## 2. Cosmetic Industry as the Research Context

Cosmetic products can be defined as any substance or mixture of natural and/or synthetic ingredients intended to be used on various external parts of the human body for the purpose of cleaning, perfuming, enhancing or changing individuals’ appearance and to maintain one’s body parts in a good condition [30]. In general, a mixture of synthetic ingredients that is based on chemical compound has greater effect on consumers’ skin and body. However, continuous usage of these chemical compounds with unlimited dosage are likely to cause bad impacts on consumers’ health, such as headaches, acne, hair problems, cancer and skin allergies in the long run [7]. Therefore, the formulation of green cosmetics using natural ingredients can substitute the cosmetics that are made of chemicals. The rising awareness about the risks that are associated with using synthetic cosmetics has shifted consumers’ preference to buy green cosmetics.

The Asian market of cosmetics has become one of the fastest growing markets [14]. In Malaysia, 210 cosmetic manufacturers are registered as homegrown cosmetic companies. Generally, the buying pattern of cosmetic products in Malaysia is not only dominated by women; men also contribute to the sales of cosmetic products [31]. In the Western region, middle-aged women are highly attracted to branded cosmetic products [32]. However, in non-Western regions, such as Hong Kong, the purchase of cosmetic products is dominant by a comparatively younger generation [31]. Thus, a further investigation on consumers’ profiles is crucial to understand Malaysian consumers’ green purchase behavior. In addition, this will help the marketing managers tailoring their promotional strategies to increase the value of shopping experiences, and segmenting the right customers. Furthermore, understanding the consumers’ profiles will broaden the horizon of extant literature to gain deeper insight on green purchase behavior, in order to profile the green consumers. 

## 3. Theoretical Framework and Hypotheses Development

### 3.1. Direct Relationships

According to the VBN theory, green purchase behavior is a function of personal norm and is linked to environmental beliefs through different types of values [15,33]. Personal norm refers to individuals’ moral obligation to engage in behavior that protects the environment, such as purchasing green cosmetic products [34]. As suggested by the VBN theory, individuals involved in positive behavior can benefit the environment. Such individuals feel that they should do the right thing (moral norm), especially when they feel responsible for the consequences of their actions towards the environment. Individuals’ awareness of consequences and ascription of responsibility are major elements of their pro-environmental beliefs that play vital role in forming personal norms [33]. Stern [33], p. 366, argued that, engaging in the behavior occurred “when an individual comes to believe that a personal value is threatened and that he or she can relieve that threat by appropriate action”. 

Value orientation is defined as a guiding principle regarding desired or appropriate states or outcomes [35], p. 15, and is expected to affect the way people formulate environmental beliefs [33]. In VBN theory, the formation of values has been modified according to the environmental movement and specified under three main value orientations that consist of the altruistic value, biospheric value and egoistic value [33]. Generally, past studies focused on these three values to examine pro-environmental behavior among green consumers [36,37,38]. However, not much attention has been given to understand the role of hedonic value in predicting consumers’ green purchase behaviors. According to Chen et al. [39], the hedonic value represents the degree to which a product/service arouses emotions and creates pleasant experiences. It is needless to say that not all consumers exhibit green purchase behavior driven by the environmental welfare motive. There might be other consumers who display green purchase behavior for the sake of a pleasant experience and also driven by their inner good feeling. Therefore, this study considers hedonic value to understand the green purchase behavior among Malaysian consumers in the context of cosmetics purchase. 

Altruistic value is defined as the feeling of concern for other people in relation to the environment [40]. Past studies have stressed the importance of considering altruistic value in the environmental studies [16]. Following this norm, the altruistic value and hedonic value are expected to have a direct influence on consumers’ pro-environmental belief to perform green behavior in the context of cosmetic purchase. Based on the above discussions, the following hypotheses are developed:

**Hypothesis 1** **(H1).** *The altruistic value positively affects pro-environmental beliefs.*

**Hypothesis 2** **(H2).** *The hedonic value positively affects pro-environmental beliefs.*

Based on VBN theory, environmental beliefs consist of three elements, namely, human–environmental relations (new environmental paradigm) which threatens the consequences of their actions (awareness of adverse consequences) and thus give signals to them to take the corrective measures (ascription of responsibility) towards the environment [33]. Pro-environmental beliefs can be described as consumers’ attitudes that display specific belief in purchasing green cosmetics as their responsibility towards environmental welfare, health and quality of life [41]. 

Past studies have investigated the notion of beliefs in relation to awareness of adverse consequences and ascription of responsibility towards personal norm in the context of climate protection [42], green curtailment behavior [43], marine protection [44] and sustainable travel mode choice [45]. Consequently, the relationship between personal norms and other pro-environmental behaviors, such as non-activist behavior in the public sphere [46], organizational behavior [47] and environmental citizenship behavior [48], are well documented. However, there is a dearth of research that measures the direct relationships between beliefs and personal norms; and personal norms and green purchase behaviors in the context of cosmetics purchasing. Guided by the VBN theory, pro-environmental beliefs are expected to have a direct influence on personal norms and in turn it may influence consumers’ green purchase behaviors of cosmetic products. Considering this, the following relationships are hypothesized:

**Hypothesis 3** **(H3).** *Pro-environmental beliefs positively affect personal norm.*

**Hypothesis 4** **(H4).** *Personal norms positively affect green purchase behavior.*

### 3.2. Gender as a Moderator 

It is argued that there is a discrepancy between what people think, believe and say and how they eventually behave [49,50]. One possible explanation for these findings may be due to the fact that, besides having positive feelings, attitude and intentions toward purchasing green products, there might be other factors that indirectly affect consumers’ final purchase decision. In order to minimize the value-belief, belief-norm and norm-behavior gaps that hinder consumers from translating their values, beliefs, inclinations and norms into actual behavior, there is a need to consider a third factor as a possible moderator to strengthen these relationships. 

It is evident that gender differs in terms of decision-making and information processing that leads to multiple buying behaviors [51]. In fact, both males and females have various buying experiences subject to their expectations and personality traits where females exert more hedonic consumption compared to males [52]. This is supported by past research indicating that the hedonic value affects shopping experiences for males and females differently [53]. Additionally, Zhang [54] argued that females perform certain roles more, compared to men, such as they tend to be more social, emotional and caring towards others (altruistic). On the other hand, men are comparatively more independent and braver than females. Contrastingly, Akehurst et al. [12] have found no significant effect of socio-demographic variables including gender, income, age and education towards green purchase behavior. 

Likewise, past studies found that, under certain circumstances, female consumers act more environmentally and are engaged in more pro-environmental behavior compared to their male counterparts [55,56]. Similarly, in a cross-cultural study, Hunter et al. [57] examined gender differences in the environmental context among 22 countries and found that female respondents exhibit more pro-environmental behavior compared to males (e.g., purchasing organic food and recycling behavior). Another stream of research revealed there is no gender differences in performing environmentally significant behavior [27,28]. Conversely, other studies found that male consumers are more likely to participate in pro-environmental behavior compared to female [25,26]. 

Based on the above discussions, it is assumed that gender can moderate all direct relationships developed in this study. Particularly, gender can moderate the relationship between value orientation and pro-environmental beliefs, pro-environmental beliefs and personal norms and personal norms and green purchase behavior in the context of cosmetic product purchase. Therefore, the following hypotheses are postulated:

**Hypothesis 5** **(H5).** *Gender moderates the relationship between altruistic values and pro-environmental beliefs.*

**Hypothesis 6** **(H6).** *Gender moderates the relationship between hedonic values and pro-environmental beliefs.*

**Hypothesis 7** **(H7).** *Gender moderates the relationship between pro-environmental beliefs and personal norms.*

**Hypothesis 8** **(H8).** *Gender moderates the relationship between personal norms and green purchase behavior.*

### 3.3. Conceptual Framework

**Figure 1:**
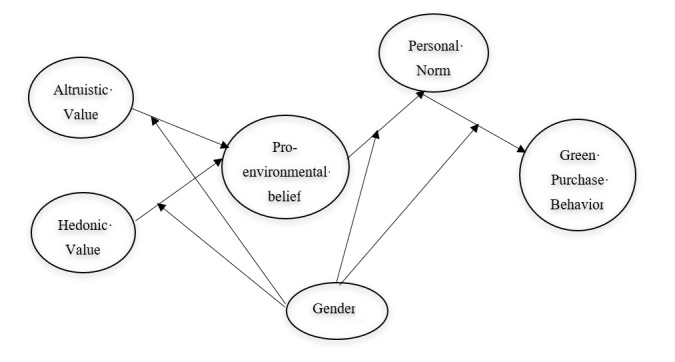
Proposed conceptual framework.

The conceptual model developed in this study is summarized in Figure 1. This model examines the relationship between values orientation and pro-environmental beliefs (H1 and H2). Moreover, it tests the effect of pro-environmental beliefs on personal norms (H3). Furthermore, it predicts the effect of personal norms on green purchase behavior (H4). In addition, it verifies the moderating role of gender upon all hypothesized direct relationships (H5 to H8).

## 4. Methodology

### 4.1. Measurement

This research employed a quantitative approach and used questionnaire survey to collect data from the respondents. All scales were borrowed from the existing literature. The green purchase behavior scale (five items) was borrowed from Khare [58], whereas the personal norm scale (threee items) was borrowed from Ghazali et al. [59]. Additionally, to measure pro-environmental beliefs a three-item scale was used, which was adapted from Kim et al. [60]. On the other hand, the altruistic value and hedonic value scales were borrowed from Izagirre-Olaizola et al. [61] and Ghazali et al. [59], respectively (Table A1). All items were rated on a five-point Likert scale, where 1 indicated “strongly disagree” and 5 indicated “strongly agree”.

### 4.2. Research Design

To achieve the research objectives, this study conducted an online survey to gather data from respondents. Use of online survey has the potential to collect a large amount of data efficiently and economically within a comparatively short time. In fact, the use of the online survey can reduce error in data collection [62]. To ensure the representativeness and adequacy of the items, the questionnaire was content-validated by three renowned professors from two reputed public universities in Malaysia. To confirm the readability and understandability of items, face validity was performed on 15 MBA and PhD students. The questionnaire was also piloted through 100 questionnaires. Based on the 83 returned responses, additional modifications were made to the arrangement of the questions and to the language used. The instrument was tested for reliability and Cronbach’s α was found satisfactory (greater than 0.70 for all constructs used in this study [63]). 

### 4.3. Sampling and Sample Size 

The non-probability judgmental sampling technique was utilized to gather data from consumers who had experience in purchasing green cosmetic products in last six months. This sampling technique was adapted since it was impossible to get a list of all elements of the population; furthermore, it permits a theoretical generalization of the findings [64]. Google Docs was used to develop the online questionnaire. The web link for the online questionnaire was then distributed via social media platforms, such as Facebook, Whatsapp and through personal contacts of the researchers. 

The required sample size was decided based on the rule of thumb suggested by Chin [65] i.e., “power analysis”. This study used G*Power to calculate the sample size based on statistical power [66]. The output of this analysis suggested a minimum sample size of 76 cases for each group to achieve power greater than 0.80. Thus, the sample size of 240 (79 male, 161 for female) was deemed appropriate since it exceeded the minimum requirement. 

### 4.4. Respondents’ Profile

The participants’ demographic characteristics, in terms of their age, income, race, marital status, and profession, are illustrated in Table 1. This sample included 79 responses from male respondents and 161 responses from female respondents. A possible explanation for this unequal distribution of gender can be ascribed to the fact that, in the Malaysian context, most of the consumers purchasing cosmetic products are female rather than male. This justification is in line with past studies which were conducted in the Malaysian context and found that while usage of green cosmetic has become a trend among both genders, female consumers showed more interest towards green cosmetics compared to male consumers (see [14,67]) However, Cheong et al. [31] suggested that in Malaysia, there is a growing demand for skincare products among men in urban areas. Nevertheless, there remains a greater demand among Malaysian female consumers for such products due to high female workforce participation (46.8%, according to the World Bank) and better education, knowledge, exposure and familiarity with green cosmetics and skincare brands. Moreover, Cheong et al. [31] found that the young female consumers, whose ages ranged between 12–35, are more likely to purchase cosmetic products, with a higher educational background, and urban dwellers who resides in middle to upper middle-income households. 

## 5. Data Analysis and Results

Structural equation modeling (SEM) with variance approach using the SmartPLS software [68] was used to examine the research model and to run the multi-group analysis (MGA). According to Reinartz et al. [69], Partial Least Squares (PLS) is the advisable approach when the main concern of a research is theory development and prediction. Moreover, PLS-SEM is a comprehensive approach that can examine all relationships between the constructs in the measurement and structural model at the same time [70]. Furthermore, PLS-SEM can handle complicated model that has direct and indirect relationship [71]. Most importantly, PLS-SEM is a nonparametric technique that is suitable for MGA [70,71]. 

The assessment of the theoretical model using SmartPLS involved evaluating the validity and reliability of the reflective measurement models, followed by estimating the structural mode in terms of in-sample explanatory power (R2), out-of-sample predictive relevance (Q2) and significance of the standardized path coefficients, as well as the model fit using the standardized root mean square residual (SRMR) [72]. After that, MGA was performed using Henseler’s MGA [70] and the permutation test [73]. In addition, measurement invariance was confirmed using Measurement Invariance of Composite Model Measurement Invariance of Composite Model (MICOM) before performing the MGA. 

### 5.1. Common Method Variance Assessment 

This study collected data using a cross sectional survey design; thus, it was necessary to examine the presence of common method variance (CMV) [74]. Harman’s single factor technique was used to achieve this objective [74]. CMV exists if one factor explains the majority of variance. The output of principle component analysis showed that the first factor explained 45.16% of the total variance. Hence, CMV was not an issue in this study. In addition, the recent method of full collinearity suggested by Kock [75] was adopted to detect the potential of CMV situations. According to Kock [75], when the values of variance inflation factor (VIF) for all construct in the structural model are less than 3.3, it is viewed as the indication of no collinearity. In this study, the VIF values of all constructs ranged between 1 and 1.843, indicating lack of collinearity issue. 

### 5.2. Measurement Model Assessment 

**Figure 2:**
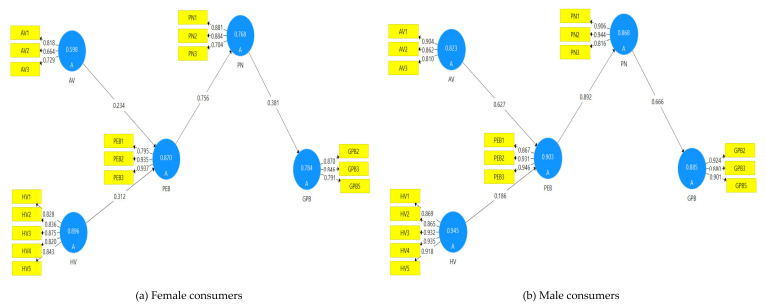
**(a)** Results of assessment of model for male consumers; **(b)** Results of assessment of model for female consumers. Note: AV = altruistic value, HV = hedonic value, PEP = pro-environmental beliefs, PN = personal norm, GPB = green purchase behavior.

First, convergent validity which refers to the extent to which a set of indicators that measure the same construct hang together positively was assessed through factor loading (FL), composite reliability (CR) and average variance extracted (AVE) [72]. The standardized values of loadings required to be greater than 0.60 [76], composite reliability should be greater than 0.70 [72], and AVE should be more than 0.50 [71]. The results in Table 2, Figure 2a,b revealed that the factor loading for all items exceeded the threshold value of 0.60, except GP1 and GP4, and thus were removed. Composite reliability for all constructs surpassed the cut-off value of 0.70, and AVE for all latent variables were above 0.50 [71]. Thus, the measurement model for the full and split data sets possessed convergent validity. 

Next, discriminant validity which indicates the extent to which each construct possesses a unique attribute that makes it different from others in the conceptual model was assessed using two methods. The first method (Fornell-Larcker criterion) involved comparing the square root of AVE for each latent variable with the correlation of other constructs in the model. The second method (the heterotrait-monotrait (HTMT)) required comparing the ratio between construct correlations to within construct correlation [77]. Table 3 and Table 4 demonstrate that the discriminant validity for full and split dataset is established. Particularly, the diagonal values (Table 3) for all constructs are greater than other values in raw and column [78] and HTMT values (Table 4) for all constructs are less than HTMT0.90 [79]. 

The assessment of the goodness of the three models (full and split) was examined using standardized root mean square residual (SRMR) [80]. SRMR value which is less than 0.08 indicates acceptable fit. The SRMR value was 0.073 for full model, 0.075 for first group (Mmale) and 0.079 for second group (female) which were below than the recommended value of 0.08 indicating a good fit between empirical covariance matrix and theoretical covariance matrix implied by the models. 

### 5.3. Structural Model 

Table 5 illustrates the hypothesis testing results for full and split dataset using the bootstrapping procedure with 5000 re-samples. The results show that altruistic value exerts positive and significant effect on perceived environmental beliefs for full and split dataset (male and female). Furthermore, the results demonstrate that hedonic value has significant positive effect on perceived environmental beliefs for the full and female dataset. However, this link was not significant for males. In addition, the results reveal that perceived environmental beliefs have a significant and positive effect on personal norm for full and split datasets. Besides, the results affirm that, personal norm exerts positive effect on green purchase behavior for the full, male and female datasets. Based on these results, it can be concluded that H1, H2, H3 and H4 are supported. 

The next step in evaluating the quality of the structural models involved calculating R2 values for endogenous constructs as indicative of the explanatory powers of the models [71]. As shown in Table 6, perceived environmental beliefs have an R2 value of 34.9% for the full model, 60.4% for males, and 24.2% for the female dataset. In addition, personal norm has an R2 value of 63.8 for the full dataset, 79.5% for the male dataset and 57.2% for females. Furthermore, green purchase behavior has an R2 value of 24.5%, 44.4% and 14.5% for the full, male and female datasets, respectively. According to Falk and Miller [81], R2 should be greater than 0.10 to reach the minimum level of explanatory power; thus, all endogenous constructs of this study for full and dataset possess explanatory power.

Finally, the predictive relevance (Q2) of all datasets was assessed using blindfolding procedure [82,83], as illustrated in Table 5. Q2 values for perceived environmental beliefs, personal norm and green purchase behavior for all dataset (full and split) were greater than zero, thus confirming the predictive relevance of all models [84]. 

### 5.4. Measurement Invariance

Before comparing the results between males and females, in terms of their values, beliefs, norms and purchasing behavior towards green products, this study performed the MICOM [72]. The main concern of this test is to make sure that the both groups have similar understanding of the measurements. Moreover, this process is mandatory before performing multi-group analysis (MGA) [72]. The MICOM procedures build on the scores of latent variables. In PLS-SEM, these latent variables are represented as composites, that is, linear combinations of indicators, and the indicator weights are estimated by the PLS-SEM algorithm [72]. The procedures of MICOM involves three steps: (i) configure invariance assessment (measurement models have the same basic factor structure for both groups); (ii) compositional invariance assessment (composite scores are not significantly different across groups); (iii) equality of composite means values and variances. If configurable and compositional variances are established, partial measurement invariance is confirmed and it is possible to compare the path coefficient across the two groups. If partial measurement invariance is established and additionally, the composite has equal mean values and variance across all group; therefore, full measurement invariance is confirmed. 

The PLS-algorithm and PLS-permutation procedures, with a 5000 re-sample and two-tail test, were performed. The results are shown in Table 2 and Table 6. First, configural invariance is established because the measurement models have the same factor structure for all constructs across males and females (Table 2). Secondly, compositional invariance is also confirmed because the composite scores for all constructs are equal across groups. Particularly, the permutation test denotes that none of the correlation (c) values is significantly different from one another. Finally, equality of mean values and variances was assessed across the two groups. The result exhibits that only personal norm have equal mean values; however, the other composite constructs have significant differences, in terms their means values, because the result falls outside the 95% confidence interval. Moreover, the result reveals that all composite constructs have equal mean values expect GPB. Based on the results of MICOM, partial measurement invariance is established (Table 6), which is a major requirement prior to perform MGA [72].

### 5.5. Multi-Group Analysis

PLS-MGA is performed to discover the difference by using Henseler’s MGA and the permutation method. The output of MGA reveals significant differences between males and females at 0.05 and 0.01, with respect to the effect of altruistic value on perceived environmental belief (H5), perceived environmental belief on personal norm (H7) and personal norm on green purchase behavior (H8) (Table 7). The findings of this study do not support a significant difference between males and females in regard to the effect of hedonic value on perceived environmental beliefs (H6) (Table 7). Both Henseler’s MGA and the permutation method confirmed the significance/non-significance of the differences in the results, which strengthened the findings of this research.

## 6. Discussion

This study aims to shed some light on the effect of gender on consumers’ green purchase behaviors. More specifically, this study examined the difference between male and female consumers in terms of their green purchase behavior of cosmetic products in the Malaysian context, and investigated the relationships between “values and pro-environmental beliefs”, “pro-environmental beliefs and personal norm” and “personal norm and green purchase behavior”. To achieve these objectives, a theoretical framework was developed based on the VBN theory and tested using PLS-SEM. The results found support for all hypothesized relationships, except H6, which is the moderating effect of gender on the link between the hedonic value and pro-environmental beliefs. The results are briefly discussed in the following paragraphs. 

The positive relationships between the altruistic value and pro-environmental beliefs were supported by the data. This result is consistent with the VBN theory, which argued that values have a direct effect on individuals’ environmental beliefs [33]. This result is in line with past studies that found positive relationship between values and environmental beliefs [48]. This finding revealed that Malaysian consumers are concerned about health and environmental welfare, and thus display strong beliefs in choosing green cosmetic products. In addition, the hypothesized relationship between the hedonic value and pro-environmental beliefs is also supported. This is in agreement with the VBN theory, which emphasized the crucial role of value as a key driver of consumers’ pro-environmental beliefs. In addition, it confirmed the importance of the hedonic value that needs to be incorporated in conventional consumption behavior, as well as green behavior studies [85]. This result indicates that Malaysian consumers perceive their experience of purchasing green cosmetic product as a source that may boost their happiness, which ultimately may motivate them to purchase these products. 

The finding of this research confirmed the positive relationship between pro-environmental beliefs and personal norms. This output is in agreement with the VBN theory. According to this theory, individuals’ beliefs about the environment will affect their norm. More specifically, consumers’ awareness of consequences and ascription of responsibilities activate their personal norm to behave in such a way that can help and protect the environment [2]. In addition, this study found support for the positive relationship between personal norm and green purchase behavior. Again, this result is consistent with VBN theory. These findings are also in agreement with past research [44]. 

This study compares the green preference between male and female consumers pertaining to the effect of values, beliefs and personal norms on their green purchase behavior. The MGA results revealed significant differences between male and female consumers’ behavioral patterns. The results suggest that males scored higher than females in these relationships. This is in line with past studies that confirmed the differences between males and females in terms of their values, attitude and behavior [26,86]. In addition, these results are consistent with past literature that found male consumers are more sensitive towards environmental principles, values and issues, resulting in more pro-environment behaviors compared to female consumers [25]. In detail, the effects of the altruistic value on pro-environmental beliefs are found stronger for males (B = 0.627), compared to females (B = 0.234), which provide support for H5. The result also revealed that the relationship between PEB and PN is higher for males (B = 0.892) compared to females (B = 0.756), which confirmed H7. In addition, the MGA’s output found that male consumers scored higher than females on the relationship between PN and GPB which provides support for H8. Contrary to the expectation, no difference was observed between males and females with respect to the effect of the hedonic value on PEB. The hedonic value positively affects PEB for females, but for males there was no significant relationship. A possible explanation for this result can be ascribed to the fact that male consumers are more rational compared to female in their consumption and purchase behaviors. More clearly, male consumers are less likely to make a purchase decision only to obtain happiness. 

Interestingly, these results contradict the gender socialization theory [86] which refers to the learning of behavior and attitudes that considered appropriate for a given sex. According to this theory, the behavior that is seen to be appropriate for each gender is largely determined by societal and cultural values in a given society [87]. For example, certain cultures emphasize masculinity more, which is typically associated with values including assertiveness, independence, rationality, heroism, task-oriented, achievement and success [88,89]. On the other hand, some cultures stress more on femininity, which is associated with values like considerateness, sensitivity, responsibility, emotion, cooperation, relationship-oriented and caring [89]. According to Hofstede’s [90] categorization of culture, Malaysia is high on masculinity and low on femininity; thus, it was expected that female participants in this study were supposed to be more engaged in purchasing green cosmetic products, because they hold stronger values and attitudes towards the environment than male participants [91]. Moreover, female consumers are more socially responsible and environmentally concerned than males [26,92]. Additionally, female consumers are likely to consider the effect of their purchasing behavior and consumption habits on others than males [93]. A possible explanation for this result can be due to the fact that culture is dynamic, flexible and changeable over time [94]. In particular, Malaysian culture has changed during the passage of time, and both males and females have become more caring and responsible about the welfare, health and safety of others. Therefore, they tend to purchase green cosmetic products that are less harmful for the health and environment. In addition, this result can be due to the lack of elements of hedonic value in cosmetic products’ designs, that make male consumers unable to recognize their feelings when purchasing the cosmetic products.

## 7. Theoretical Contribution and Managerial Implications

From a theoretical point of view, this research contributes significantly to the existing body of knowledge. First, this is relatively a new study that extended the theory of VBN by bringing together self-transcendence value (altruistic value) and self-enhancement value (hedonic value). The consideration of the hedonic value provides further insights to the existing literature, where it addresses the gap of environmental behavior based on the emotional feeling of consumers. Second, this study contributes to the literature by introducing new relationships; particularly, the link between the hedonic value and PEB, the moderating role of gender on the relationships between (i) values (hedonic and altruistic) and pro-environmental beliefs, (ii) pro-environmental beliefs and personal norms and (iii) personal norms and green purchase behavior in the context of cosmetic purchase. The consideration of gender as a moderator on the existing links is specifically to gain further insights on the pattern of buying behavior from different consumers’ background and profile. These findings provide useful information to marketers to analyze the buying patterns of the consumers in order to predict the future trends in consumers’ purchasing decisions [14]. Finally, yet importantly, this study confirmed the suitability of VBN theory in explaining consumer behavior in non-Western cultural contexts, including Malaysia. 

Practically, the findings of this research provide a clear understanding on the buying pattern from different gender groups. Particularly, male consumers exert stronger altruistic values than female consumers in purchasing cosmetic products. Thus, it is suggested for the marketers to develop green marketing campaigns that target the female consumer segment. This will encourage the interest of female consumers to get involved in these campaigns and, indirectly, will persuade them to purchase green products. Engaging in values is beneficial to the managers in cosmetic industry since it contributes to the attaining of loyalty and satisfaction among consumers [95]. Furthermore, these findings demonstrate the important role of altruistic and hedonic values in influencing consumers’ beliefs that lead to their purchasing decision on green cosmetic products. Thus, these findings are useful for marketers aiming to instill the elements of altruistic and hedonic values in their advertisements that emphasize the benefits of green cosmetics: not only for their bodies, but also for beneficial conserving of the environment. Another relevant strategy to increase the confidence of consumers toward green products is through adapting the element of the hedonic value in products’ promotional strategies. For example, offering product membership to new customers so that they can enjoy the point-of-purchase and points redemption in the next purchase. Most importantly, the output of this study found significant differences between the male and female cohorts in terms of their attitudes and behaviors towards green cosmetic products. Male consumers tend to purchase this category of products more than female consumers. Therefore, it is the responsibility of the manufacturers, marketers and policy makers to produce and promote these products for both male and female segments.

## 8. Limitations and Future Research Directions

Although this study has its merits in providing useful insights about the role of gender as a moderator, it is not beyond of some limitations. However, the limitations addressed in this research may provide directions for the future research. For example, the present study used a cross sectional survey design to collect data, whereas future studies can consider a longitudinal design to tap into behavioral changes appropriately. Furthermore, this study considered gender as the moderator on values, beliefs, norms and behavior relationships. Hence, it is suggested that future studies can consider other demographic factors as moderators, such as age, education and income level, to gain more insights on consumers’ purchase behaviors. Examining other demographic elements of consumers may provide useful information for marketers to project different strategies for different market segments.

## Figures and Tables

**Table 1:** Demographic profile.

Valuables	Full Sample (*n* = 240)	Male (*n* = 79)	Female (*n* = 161)
	Frequency	Frequency	Frequency
Age			
35 and less	171	49	122
More than 36	69	30	39
Income			
RM3500 and less	131	38	93
RM3501 and above	109	41	68
Race			
Malay	150	55	95
Chinese	60	10	50
Indian	30	12	18
Marital Status			
Single	79	23	56
Married	161	56	105

**Table 2:** Assessment of full measurement model and samples.

Full Dataset (*n* = 240)					Male (*n* = 79)			Female (*n* = 161)		
Construct	Items	Loadings	CR	AVE	Loadings	CR	AVE	Loadings	CR	AVE
AV	AV1	0.854	0.824	0.61	0.904	0.894	0.739	0.818	0.782	0.547
	AV2	0.751			0.862			0.664		
	AV3	0.733			0.81			0.729		
GPB	GPB2	0.894	0.897	0.744	0.924	0.929	0.813	0.87	0.874	0.699
	GPB3	0.864			0.88			0.846		
	GPB5	0.828			0.901			0.791		
HV	HV1	0.841	0.935	0.743	0.869	0.957	0.818	0.828	0.923	0.707
	HV2	0.844			0.865			0.836		
	HV3	0.893			0.932			0.875		
	HV4	0.862			0.935			0.82		
	HV5	0.869			0.918			0.843		
PEB	PEB1	0.823	0.928	0.811	0.867	0.939	0.838	0.795	0.92	0.795
	PEB2	0.934			0.931			0.935		
	PEB3	0.94			0.946			0.937		
PN	PN1	0.886	0.882	0.716	0.906	0.92	0.793	0.881	0.865	0.684
	PN2	0.903			0.944			0.884		
	PN3	0.74			0.816			0.704		

Note 1. Items GP1, and GP4 removed from full and split dataset due to low loading (<0.60). Note 2. CR = Composite reliability, AVE = Average variance extracted.

**Table 3:** Discriminate validity (Fronell–Larcker criterion).

Full Dataset (*n* = 240)						Male (*n* = 79)					Female (*n* = 161)				
Construct	AV	GPB	HV	PEB	PN	AV	GPB	HV	PEB	PN	AV	GPB	HV	PEB	PN
AV	**0.781**					**0.86**					**0.74**				
GPB	0.615	**0.862**				0.676	**0.902**				0.549	**0.836**			
HV	0.676	0.532	**0.862**			0.756	0.58	**0.904**			0.621	0.5	**0.841**		
PEB	0.552	0.428	0.528	**0.9**		0.767	0.601	0.66	**0.915**		0.427	0.294	0.457	**0.892**	
PN	0.619	0.495	0.657	0.798	**0.846**	0.766	0.666	0.715	0.892	**0.89**	0.537	0.381	0.624	0.756	**0.827**

Note. Diagonal values in bold represent square root of AVE; other values represent the correlation between constructs.

**Table 4:** Discriminant validity (HTMT 0.90 Criteria).

Full Dataset (*n* = 240)						Male (*n* = 79)					Female (*n* = 161)				
Construct	AV	GPB	HV	PEB	PN	AV	GPB	HV	PEB	PN	AV	GPB	HV	PEB	PN
AV															
GPB	0.826					0.79					0.837				
HV	0.868	0.613				0.845	0.625				0.874	0.601			
PEB	0.692	0.493	0.582			0.864	0.667	0.698			0.556	0.348	0.51		
PN	0.845	0.618	0.794	0.871		0.851	0.759	0.788	0.881		0.805	0.516	0.796	0.88	

**Table 5:** Assessment of structural model.

Full Sample (*n* = 240)							Male (*n* = 79)					Female (*n* = 161)				
Path		Std Beta	SE	t-Values	R^2^	Q^2^	Std Beta	SE	t-Values	R^2^	Q^2^	Std Beta	SE	t-Values	R^2^	Q^2^
H1	AV→PEB	0.359	0.086	4.157 **	0.349	0.263	0.627	0.135	4.658 **	0.604	0.47	0.234	0.101	2.303 *	0.242	0.158
H2	HV→PEB	0.286	0.086	3.305 **			0.186	0.119	1.563			0.312	0.104	2.987 **		
H3	PEB→PN	0.798	0.025	32.021 **	0.638	0.423	0.892	0.024	36.712 **	0.795	0.593	0.756	0.033	22.711 **	0.572	0.369
H4	PN→GPB	0.495	0.048	10.221 **	0.245	0.171	0.666	0.058	11.418 **	0.444	0.336	0.381	0.076	5.042 **	0.145	0.093

Note: * *p* < 0.05; ** *p* < 0.001.

**Table 6:** Measurement invariance result using permutation test.

Compositional Invariance Correlation = 1					Equal Mean Assessment			Equal Variance Assessment			
Construct	Configure Invariance	C = 1	95% CI	Partial Measurement Invariance Established	Difference of Mean Value	95% Confidence Interval	Equal Mean	Difference of the Variances Value	95%CI	Equal Variance	Full Measurement Invariance Established
AV	Yes	0.994	0.983–1.000	Yes	−0.268	−0.234–0.228	No	0.234	−0.277–0.249	Yes	No
GPB	Yes	1	0.993–1.000	Yes	−0.33	−0.229–0.236	No	0.611	−0.304–0.272	No	No
HV	Yes	0.999	0.997–1.000	Yes	−0.25	−0.221–0.229	No	0.065	−0.298–0.26	Yes	No
PEB	Yes	0.999	0.999–1.000	Yes	−0.25	−0.224–0.216	No	0.076	−0.27–0.257	Yes	No
PN	Yes	0.999	0.997–1.000	Yes	−0.107	−0.231–0.227	Yes	0.034	−0.238–0.223	Yes	Yes

**Table 7:** Assessment of group difference.

Hypotheses	Relationship	Std Beta Values		SE Values		t-Values		Path Coefficient Differences	*p*-Values		Supported
		Male	Female	Male	Female	Male	Female		Henseler MGA	Permutation	
H5	AV→PEB	0.627	0.234	0.135	0.234	4.658 **	2.303 *	0.393	0.018 *	0.045 *	Yes/yes
H6	HV→PEB	0.186	0.312	0.119	0.312	1.563	2.987 **	−0.125	0.442	0.499	No/no
H7	PEB→PN	0.892	0.756	0.024	0.756	36.712 **	22.711 **	0.136	0.001 *	0.011 *	Yes/yes
H8	PN→GPB	0.666	0.381	0.058	0.381	11.418 **	5.042 **	0.285	0.003 **	0.008 **	Yes/yes

Note: * *p* < 0.05, ** *p* < 0.01

## Appendix A

**Table A1:** Survey items for the constructs.

Altruistic Value	Numbers	Content
	AV1	I always consider the health aspects of my cosmetic purchases.
	AV2	While purchasing cosmetic products, I focus on environmentally friendly cosmetics.
	AV3	It concerns me that people consume high chemical cosmetics with negative environmental impact.
**Hedonic Value**	HV1	Buying organic cosmetics give me pleasure.
	HV2	Buying green cosmetics make me feel like doing morally the right thing.
	HV3	The use of green cosmetics can affect my well-being positively.
	HV4	I enjoy using green cosmetics.
	HV5	I feel relaxed using green cosmetics.
**Pro-environmental Belief**	PEB1	I am willing to participate in preserving the environment.
	BEB2	I believe that having sense of responsibility towards environmental problems is important.
	BEV3	I believe that the moral obligation to help the environment is important.
**Personal Norm**	PN1	I feel obliged to save environment where possible.
	PN2	I should do what I can to conserve natural resources.
	PN3	I feel a strong personal obligation to use green cosmetics.
**Green Purchase Behavior**	GPB1	I usually prefer to purchase cosmetic products with reusable packaging (e.g., reusable glass bottle for cream and cleanser products).
	GPB2	Whenever I need to buy cosmetic products, I always purchase cosmetic products with no chemical ingredients.
	GPB3	I try to purchase cosmetic products that are free from chemical even though they are more expensive.
	GPB4	I always purchase biodegradable cosmetic products (which can be easily disposed after use).
	GPB5	I always refrain myself from purchasing cosmetic products with chemical ingredients.
